# Determinants of survival in very low birth weight neonates in a public sector hospital in Johannesburg

**DOI:** 10.1186/1471-2431-10-30

**Published:** 2010-05-06

**Authors:** Daynia E Ballot, Tobias F Chirwa, Peter A Cooper

**Affiliations:** 1 Department of Paediatrics, University of the Witwatersrand Medical School, York Road, Parktown, Johannesburg, South Africa; 2 Epidemiology and Biostatistics Division, School of Public Health, University of the Witwatersrand Medical School, York Road, Parktown, Johannesburg, South Africa

## Abstract

**Background:**

Audit of disease and mortality patterns provides essential information for health budgeting and planning, as well as a benchmark for comparison. Neonatal mortality accounts for about 1/3 of deaths < 5 years of age and very low birth weight (VLBW) mortality for approximately 1/3 of neonatal mortality. Intervention programs must be based on reliable statistics applicable to the local setting; First World data cannot be used in a Third World setting. Many neonatal units participate in the Vermont Oxford Network (VON); limited resources prevent a significant number of large neonatal units from developing countries taking part, hence data from such units is lacking. The purpose of this study was to provide reliable, recent statistics relevant to a developing African country, useful for guiding neonatal interventions in that setting.

**Methods:**

This was a retrospective chart review of 474 VLBW infants admitted within 24 hours of birth, between 1 July 2006 and 30 June 2007, to the neonatal unit of Charlotte Maxeke Johannesburg Academic Hospital (CMJAH) in Johannesburg, South Africa. Binary outcome logistic regression on individual variables and multiple logistic regression was done to identify those factors determining survival.

**Results:**

Overall survival was 70.5%. Survival of infants below 1001 grams birth weight was 34.9% compared to 85.8% for those between 1001 and 1500 grams at birth. The main determinant of survival was birth weight with an adjusted survival odds ratio of 23.44 (95% CI: 11.22 - 49.00) for babies weighing between 1001 and 1500 grams compared to those weighing below 1001 grams. Other predictors of survival were gender (OR 3. 21; 95% CI 1.6 - 6.3), birth before arrival at the hospital (BBA) (OR 0.23; 95% CI: 0.08 - 0.69), necrotising enterocolitis (NEC) (OR 0.06; 95% CI: 0.02 - 0.20), hypotension (OR 0.05; 95% CI 0.01 - 0.21) and nasal continuous positive airways pressure (NCPAP) (OR 4.58; 95% CI 1.58 - 13.31).

**Conclusions:**

Survival rates compare favourably with other developing countries, but can be improved; especially in infants < 1001 grams birth weight. Resources need to be allocated to preventing the birth of VLBW babies outside hospital, early neonatal resuscitation, provision of NCPAP and prevention of NEC.

## Background

The fourth Millennium Development Goal is to reduce the mortality of children under the age of 5 years by two thirds, before the year 2015. Neonatal mortality accounts for 37% of deaths below the age of 5 years and "improved neonatal and maternal care could save the lives of countless newborns" [1]. In the Perinatal Problem Identification Program (PPIP) http://www.ppip.co.za, a self reporting data base that covers about 40% of births in South Africa, the early neonatal mortality rate has been static over the past few years at about 9.5 per 1000 live births [2]. However, the majority of neonatal deaths remain unaudited and the national figure is thus probably higher. Very low birth weight (VLBW) infants represent a vulnerable group of newborns with a high mortality rate. There are many reports of factors affecting early survival of VLBW infants; these are summarized in Table 1[3-22]. The survival rate of VLBW infants worldwide ranges between 43% in developing countries such as Jamaica [21] to more than 90% in developed countries, such as the Netherlands [7], with an average of about 73% (See Table 1). The mortality rate for VLBW infants in Soweto, Johannesburg, between 2000 and 2002 was reported at 71% [22], which corresponds to developed countries in the mid 1980's (see Table 1).

**Table 1:** Survival of Very Low Birth Weight infants

*Location*	*Weight (Main inclusion criteria)*	*Time Period*	*Number of babies*	*Survival*	*Factors associated with survival of babies*	Reference
Texas	VLBW	1977 1995		50% 81%	Number of babies offered mechanical ventilation; Black females survival advantage	[3]

Israel	VLBW	1985-87	69	70%		[4]

Italy	VLBW ELBW	1987-1988	634	77% 44%	Lower Birth weight or gestational age, Gender, No antenatal steroids, 1 minute Apgar, No spontaneous respiration in delivery room, body temperature/pH on admission	[5]

Malaysia	VLBW	1989-1990	329	40%	Lower birth weight/gestational age	[6]

Netherlands	VLBW	1983 1995	1388 2006	75% 90%	Delivery in tertiary centre, prolonged artificial ventilation, Caesarean delivery	[7]

Taiwan	VLBW	1995-1998	162	78.4%		[8]

South America	VLBW	1997-1998	385 (11 neonatal units)	73% (49-89%)	Birth weight, gestational age, No antenatal steroids, air leaks	[9]

Sofia (Bulgaria)	VLBW ELBW	1998-1999	122 61	86% 54%	Birth weight, gestational age, low Apgar scores, Cord Ph < 7.1, Need for cardiac compressions/adrenaline in delivery room	[10]

New Zealand	VLBW	1986 1998	413 1084	81% 90.3%	Delivery in tertiary centre, No antenatal steroids	[11]

Thailand	VLBW ELBW	1996	613	76% 49%	Gestational age, birth weight, delivery room resuscitation, Pneumothorax	[12]

East Anglia (England)	VLBW	1993-1997	1244	75-79%	Gestational age, Birth weight, No antenatal steroids	[13]

USA (NICHD)	VLBW	1995-1996 1997-2002		84% 85%		[14]

Turkey	VLBW	1997-2002	122	84%	Birth Weight	[15]

Spain	VLBW	2002 2005	8942	80.6% 84.8%	Outborn, birthweight	[16]

India	VLBW	3 years (not stated)	260	63%	Birth weight, gestational age, maternal bleed, 1 minute Apgar, apnoea, neonatal septicaemia, shock, hypothermia, no antenatal steroids	[17]

Brazil	VLBW	2004/5	579 (16 tertiary units)	84% (69-95%)	Gestational age, maternal hypertension, 5 minute Apgar, respiratory distress, place of birth	[18]

Malaysia	VLBW	1993 2003	69 60	62,3% 81,6%		[19]

Thailand	VLBW ELBW	2002/3	78	81% 52%	Birth weight, gestational age, congenital anomalies, No antenatal steroids, 5 minute Apgar, intubation in delivery room, respiratory distress syndrome	[20]

Jamaica	ELBW	2002/3	47	43%	Gestational age, birth weight, gender, No antenatal steroids, Caesarean delivery	[21]

South Africa	VLBW ELBW	2000-2002	2164	71% 32%	Birth weight, 1 & 5 minute Apgars, Caesarean delivery, antenatal care, gender	[22]

VLBW = Very low birth weight (< 1501 grams) ELBW = extremely low birth weight (< 1001 grams)

There has been steady improvement in the overall early survival of VLBW infants over time (see Table 1) e.g. from 50% in 1977 to 81% in 1995 in Texas [3] and from 81% in 1986 to 90.3% in 1998 in New Zealand [11]. Malaysia, a developing country, showed a similar improvement from 62% in 1993 to 81.6% in 2003 [19]. The degree of improvement, however, is less marked in more recent years - the NICHD showed almost no improvement in early VLBW survival between 1995/6 (84%) and 1997 - 2002 (85%) [14].

Audit of neonatal care by participating in a database such as the Vermont Oxford network (VON) http://www.vtoxford.org assists quality control provides a benchmark for comparison and opportunities for research and collaboration with other neonatal units. In developing countries with busy, under-resourced neonatal units, participation in the VON is difficult as it requires appropriate information systems and additional dedicated staff members. There is therefore a lack of current, valid statistics from such units, even though large numbers of patients are treated annually. It is essential to have this information to guide forward planning for therapeutic interventions, budgeting and staffing, with the aim of improving outcome. Local data relevant to a developing country is essential to facilitate this planning; it is not possible to transpose data from one area to another.

The purpose of this study was to review the survival to hospital discharge and morbidity of VLBW infants at Charlotte Maxeke Johannesburg Academic Hospital (CMJAH), a busy neonatal unit in a developing country.

## Methods

This was a retrospective record review of all neonates with a birth weight < 1501 grams admitted to the neonatal unit of CMJAH within 24 hours of birth from 1 July 2006 to 31 June 2007. All inborn neonates were admitted directly to a labour ward nursery, so statistics included inborn babies who died shortly after birth. VLBW babies who were delivered at outlying primary level hospitals or clinics and those who were born before arrival in hospital (BBA) were also admitted to the neonatal unit. Data was entered from hospital records onto a Microsoft Access (2003) database. Maternal information obtained from the delivery records included age, parity, gravidity, antenatal care, administration of antenatal steroids, syphilis screening and treatment, human immunodeficiency virus (HIV) screening and prophylaxis, place of delivery, fetal presentation and mode of delivery. HIV screening followed a protocol of voluntary counseling and testing; mothers could refuse to be tested. Prophylaxis was only given to infants where mothers were proven to be HIV positive. Polymerase chain reaction (PCR) testing to confirm HIV infection in the neonate was only done from 6 weeks of chronological age. Neonatal intensive care unit (NICU) admission was not determined by HIV exposure.

The baby's weight, Apgar scores and details of delivery room resuscitation were also obtained from the delivery records. Gestational age was determined from a combination of maternal history (expected date of delivery, height of fundus, first trimester ultrasound) and the Ballard score, which was done by attending clinical staff. The birth weight was plotted on Fenton [23] growth charts to determine whether the baby was appropriate for gestational age (AGA), small for gestational age (SGA) or large for gestational age (LGA). Information was available on all patients until hospital discharge. Neonatal records were reviewed by the primary author (DEB) and the final diagnoses assigned by the attending clinical staff were confirmed using the available clinical information and results of investigations. The neonatal information included duration of hospital stay, respiratory diagnosis (including hyaline membrane disease (HMD)), duration of oxygen therapy, pneumothorax, neonatal jaundice (NNJ), phototherapy, exchange transfusion, patent ductus arteriousus (PDA) and treatment, necrotizing enterocolitis (NEC) and management, intraventricular haemorrhage (IVH) and grade, periventricular leukomalacia (PVL), hypotension, infection and causative organism blood results, retinopathy of prematurity (ROP), bronchopulmonary dysplasia (BPD) (defined as oxygen requirement at 28 days of age), congenital anomalies, whether KMC was done and final outcome (discharge or death). IVH was graded according to Papile [24] the diagnosis of NEC was given if the baby had modified Bell's stage 2 or 3 [25]; ROP was diagnosed by an ophthalmologist; PDA was confirmed on echocardiogram by a paediatric cardiologist.

The cause of death was reviewed by the primary author (DEB) and classified according to the PPIP classification http://www.ppip.co.za. The PPIP was established in 1999 in South Africa as a national tool for perinatal death audit. In order to have manageable data, the *single most likely *cause of death is listed - major categories include prematurity, asphyxia, infection and congenital anomaly. Each category is further subdivided into sub-categories; prematurity is subdivided into extreme immaturity, HMD, IVH, NEC and pulmonary haemorrhage. No postmortem examinations were done on the study patients. Details of ICU admissions include indication for ventilation, dates and type of ventilatory support, (IPPV or NCPAP) and surfactant therapy. Babies who received both NCPAP and IPPV were classified as needing ventilator assistance for the purposes of analysis.

Babies were managed according to the unit policies at the time. Ventilatory support was offered to babies above 900 grams birth weight, due to severely limited tertiary resources. Babies were not routinely intubated or given NCPAP in the delivery room; ventilatory support (including NCPAP) was commenced when the infant showed signs of respiratory failure. All babies, irrespective of birth weight, were provided with standard neonatal care (nursed in an incubator, given supplemental oxygen, intravenous fluids, antibiotic therapy, blood transfusion, phototherapy as needed and KMC). Surfactant therapy was only given as rescue therapy to babies on ventilatory support, usually to those patients who did not wean rapidly from supplementary oxygen. A second dose of surfactant could be given if the baby had responded to the initial dose and then deteriorated again. NCPAP was introduced to the neonatal unit March 2006. During the period of the study, there was no rooming in facility, so mothers could only do KMC intermittently during the day. KMC was introduced once a baby was in room air and tolerating full enteral feeds. Cranial ultrasound was done during the first week of life by a paediatric neurologist and, if indicated, repeated after 1 to 2 weeks and just prior to discharge. Babies who died within the first 72 hours may not have undergone a cranial ultrasound. Screening for retinopathy of prematurity was done by an ophthalmologist at 36 weeks post conceptional age. If babies were discharged prior to this age, an outpatient appointment was booked for the ophthalmology clinic. Babies were discharged home once they had established enteral feeds, were off supplemental oxygen, maintaining temperature and had achieved a weight of 1600 grams. Some babies were discharged to regional step down facilities for weight gain, close to the time of discharge home.

### Statistical analysis

Statistical analysis was done on a personal computer using SPSS version 17 (SPSS Inc. http://www.spss.com). Continuous variables were summarised using mean and 95% confidence intervals, while categorical variables were summarised as ratios and percentages. For the purposes of analysis, babies transferred out and those discharged home directly were combined as "survivors" and compared to those babies that died during their hospital admission. Cross-tabulations of categorical variables with survival were produced and statistical associations between these categorical variables and survival outcome were done using the Chi-Square test of association. Normally distributed continuous variables were compared using the unpaired t test and the Mann-Whitney U test was used to compare discrete variables and those continuous variables that were not normally distributed. Binary outcome logistic regression was done on individual variables to predict survival. Those variables which were significant at the univariate analysis were entered into a multiple logistic regression using the backward selection procedure. All the statistical tests were conducted at 5% significance level.

### Ethics

The study was approved by the ethics committee of the University of the Witwatersrand for research on human subjects.

## Results

Among the four hundred and eighty eight eligible VLBW babies who were admitted during the study period, 474 records (97.1%) of VLBW babies born to 448 mothers were retrieved and available for review.

The overall survival was 334/474 (70.5%). The mean birth weight was 1133.5 grams (95% CI 1111.9 - 1155 .0), mean gestational age was 29.9 weeks (95% CI 29.6 - 30.1) and mean duration of hospitalisation was 25.8 days (95% CI 23.8 - 27.8). The mean age at time of death was 5.77 days (95% CI 3.66 - 7.88) and of discharge/transfer was 34.23 days (95% CI 32.13 - 36.32). The mean duration of supplemental oxygen was 8.2 days (95% CI 6.8 - 9.7) and mean duration of mechanical ventilation was 8.08 days (95% CI 6.15 - 10.01).

### Birth weight and gestational age

**Figure 1:**
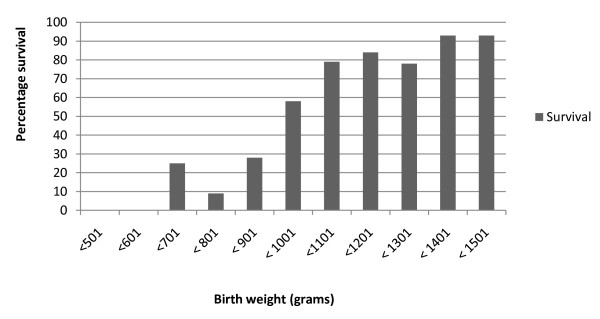
**Survival by birthweight category**.

The mean birth weight of survivors (1213 grams; 95% CI: 1192.5 - 1234.1) was significantly greater (p < 0.001) than that for babies that died (942.5 grams; 95% CI: 904.5 - 980.5). The mean gestation period for survivors (30.7 weeks, 95% CI: 30.4 - 31.0) was significantly more advanced than that of those who died (27.6 weeks, 95% CI: 27.2 - 28.1). The median 5 minute Apgar score of those babies that survived, 8 (IQR: 1-9) was significantly higher than the non survivors, 6 (IQR: 1-10) with p = 0.005. Mortality by birth weight category is shown in figure 1. As survival seem to increase with birth weight, an association between a quadratic term was fitted which also showed significant association (p=0.010) with survival.

Survival was closely related to birth weight category - ranging from zero below 601 grams to 62% (32/52) from 901 to 1000 grams and 93% (67/72) from 1301 to 1500 grams. The survival of extremely low birth weight infants (< 1001 grams) was 34.9% (50/143) compared to 85.8% (284/331) for babies with a birth weight from 1001 to 1500 grams. The adjusted survival odds ratio was 23.44 (95% CI: 11.22 - 49.00) for babies weighing from 1001 to 1500 grams compared to those weighing below 1001 grams.

The main cause of death according to the PIPP classification is shown in Table 2. The single most common cause of death was extreme multi-organ immaturity, in 40% of cases, followed by HMD in 15% of cases.

**Table 2:** PPIP classification of mortality

*Cause of death*	*Cases*	%
**Extreme mulitorgan immaturity**	56	40
**HMD**	22	15.7
**Asphyxia**	17	12.1
**NEC**	14	10
**Nosocomial sepsis**	14	10
**Septicaemia**	3	2.1
**Congenital infection**	2	1.4
**IVH**	3	2.1
**Congenital abnormality**	4	2.8
**Pulmonary Haemorrhage**	2	1.4
**Unspecified**	3	2.1

### Maternal and delivery period

The mean maternal age was 26.5 years (95% CI: 25.8 - 27.1) and 37.3% (152/407) were primiparous. Risk factors for mortality related to antenatal care, labour and delivery are presented in Table 3. Emergency Caesarean section (CS) was done for fetal distress in 63/229 (27.5%) of cases. Where emergency CS was done for maternal indications, the most common reasons were pregnancy induced hypertension 45.6% (63/138) followed by ante partum haemorrhage in 13% (18/138). No CS was done for HIV infection alone.

**Table 3:** Obstetric related risk factors and prediction of mortality

Variable (Valid cases)		Category total	Survived (%)	Died (%)	P value	Odds ratio	95% CI of OR
**SGA (461)**	Yes	178	143 (80)	35 (20)	0.001	2.07	1.32 - 3.22
	No	283	188 (66)	95 (34)			
**Gender (470)**	Female	251	192 (76.5)	59 (23.5)	0.004	1.8	1.24 - 2.69
	Male	219	141 (64.4)	78 (35.6)			
**Antenatal care (474)**	Yes	256	197 (77)	59 (23)	0.001	1.97	1.32 - 2.95
	No	218	137 (63)	81 (37)			
**Antenatal corticosteroids (474)**	Given	173	131 (75.4)	42 (24.3)	0.057	1.51	0.99 - 2.30
	Not given	301	203 (67.4)	98 (32.6)			
**HIV exposed (328)**	Yes	120	84 (70)	36 (30)	0.057	0.61	0.36 - 1.02
	No	208	165 (80)	43 (21)			
**Syphilis Exposed (474)**	Yes	12	7 (58.4)	5 (42)	0.351	0.58	0.18 - 1.85
	No	462	327 (71)	135 (29)			
**Place of birth (470)**					< 0.001		
	Inborn	383	286 (74.7)	97 (25.3)		1	
	BBA	51	20 (39)	31 (61)		0.22	0.12 - 0.40
	Out born	36	24 (66)	12 (33)		0.68	0.33 - 1.41
**Mode of delivery (464)**					< 0.001		
	NVD	197	123 (62.4)	74 (37.6)		1	
	Vaginal Breech	25	13 (52)	12 (48)		0.65	0.28 - 1.50
	Elective CS	12	9 (75)	3 (25)		1.80	0.47 - 6.88
	Emergency CS	229	182 (79.5)	47 (20.5)		2.33	1.51 - 3.59
**Presentation (448)**	Vertex	401	290 (72.3)	111 (27.7)	0.541	0.82	0.43 - 1.57
	Breech	47	32 (68.11)	15 (31.9)			
**Resuscitation at birth (474)**	Yes	154	91 (59.1)	63 (40.9)	< 0.001	0.46	0.30 - 0.69
	No	320	243 (75.9)	77 (24.1)			
**Hypothermia at birth (474)**	Yes	13	5 (38.5)	8 (61.5)	0.01	0.25	0.08 - 0.78
	No	461	329 (71.4)	132 (28.6)			
**5 minute APGAR Score (409)**	Score >= 6	304	231 (76)	73 (24)	0.093	1.52	0.93 - 2.46
	Score < 6	105	71 (67.6)	34 (32.4)			

Note: Valid cases = those with no missing data, thus, a complete case analysis

Percentages are reported for rows

Odds ratios with 95% confidence intervals are presented for each risk factor. Significant predictors of survival on univariate analysis were size for gestational age, gender, antenatal care, place of delivery, mode of delivery, the need for delivery room resuscitation and hypothermia at birth. Maternal HIV exposure, maternal infection with syphilis, the administration of antenatal steroids and the presenting part at delivery did not predict survival. Only 69% of mothers had known HIV status and 36.5% of mothers had received antenatal steroids.

### Neonatal period

Air leak was recorded in 3 patients (0.6%) and high frequency ventilation was used in 3 patients (0.6%). These variables were not included in the analysis due to small numbers. Bronchopulmonary dysplasia (BPD) (defined as oxygen requirement > 28 days) was present in 42 (8.8%) of babies. Nevirapine prophylaxis was given to 97/120 (80.8%) of the HIV exposed infants; 25/354 (7%) of mothers offered HIV testing refused consent. 11/474 (2.3%) of the babies were put up for adoption. Only 87 (18.3%) of the babies were screened for ROP prior to discharge-only two babies had ROP, both of which were stage 1. KMC was done by 211/474 (44.5%) of mothers.

Odds ratios and 95% confidence intervals for each risk factor related to the neonatal period (both disease and treatment) are shown in Table 4. HMD, NCPAP, surfactant therapy, hypotension, NEC and IVH were all predictive of survival. The need for mechanical ventilation, PDA and the presence of sepsis did not predict survival.

**Table 4:** Risk factors for mortality related to disease/treatment in the neonatal period

Risk factor (Valid cases)		Total	Survived (%)	Died (%)	P Value	Odds ratio	95% CI of Odds Ratio
**HMD (437)**	Yes	299	190 (63.5)	109 (36.5)	< 0.001	0.33	0.20 - 0.55
	No	138	116 (84.1)	22 (15.9)			
**Mechanical ventilation (474)**	Yes	99	71 (71.7)	28 (28.3)	0.76	1.08	0.66 - 1.76
	No	375	263 (70.1)	112 (29.9)			
**Nasal CPAP (474)**	Yes	96	80 (83.3)	16 (16.7)	0.002	2.44	1.37 - 4.35
	No	378	254 (67.2)	124 (32.8)			
**Surfactant therapy (474)**	Yes	90	73 (81)	17 (19)	0.014	2.02	1.15 - 3.58
	No	384	261 (68)	123 (32)			
**Sepsis (445)**	Yes	62	40 (64.5)	22 (35.5)	0.085	0.61	0.34 - 1.08
	No	383	287 (74.9)	96 (25.1)			
**Gram Negative (474)**	Yes	37	21 (56.8)	16 (43.2)	0.057	0.52	0.26 - 1.03
	No	437	313 (71.6)	124 (28.4)			
**Gram Positive (474)**	Yes	28	23 (82.1)	5 (17.9)	0.163	1.99	0.74 - 5.36
	No	446	311 (69.7)	135 (30.3)			
**PDA (474)**	Yes	26	18 (69)	8 (31)	0.887	0.94	0.4 - 2.21
	No	448	316 (71)	132 (29)			
**Hypotension (474)**	Yes	23	7 (30)	16 (70)	< 0.001	0.166	0.07 - 0.41
	No	451	327 (73)	124 (27)			
**NEC grade 2/3 (474)**	Yes	26	9 (35)	17 (65)	< 0.001	0.2	0.09 - 0.46
	No	448	325 (73)	123 (27)			
**IVH (328)**					0.004		
	No	253	209 (83)	44 (17)		1	
	Gr 1	11	8 (72.7)	3 (27.3)		0.56	0.14 - 2.20
	Gr 2	40	25 (62.5)	15 (37.5)		0.35	0.17 - 0.72
	Gr 3	16	12 (75)	4 (25)		0.63	0.19 - 2.05
	Gr 4	4	1 (25)	3 (75)		0.07	0.01 - 0.69
	PVL	4	2 (50)	2 (50)		0.21	0.03 - 1.53

Note: Valid cases = those with no missing data

Percentages are reported for rows

### Multivariate analysis

Multivariate logistic regression is shown in Table 5 for complete cases i.e. a complete case analysis with the entered variables defining a complete case. Variables entered into the model included birth weight, SGA, gender, antenatal care, place of birth, mode of delivery, NEC grade 2/3, hypotension, HMD, resuscitation at birth, hypothermia, surfactant therapy and NCPAP. Gestational age was not included in the model as it is highly correlated with birth weight (correlation coefficient 0.717 p < 0.001) and birth weight is more accurate in our setting than estimation of gestational age. IVH was not included due to the large number of missing variables. The final model showed that birth weight, gender, resuscitation at birth, BBA, hypotension, definite NEC and provision of NCPAP were significant predictors of mortality in this population. The model predicted mortality correctly in 87% of cases. Birth weight was the single most important predictor of mortality, correctly predicting mortality in 82.9% of cases. The odds ratio for death for birth weight ≤ 1001 grams was 10.41 (95% CI 6.62 to 16.6) and for gestational age< 28 weeks was 11.97 (95% CI 7.1 - 20.1).

**Table 5:** Multivariate logistic regression analysis with adjusted estimates of Odds Ratio (95% CI).

Variable	Odds Ratio	95% Confidence Interval	P-Value
Birth Weight	1.008	1.006 - 1.01	< 0.001
**Gender**			
Male	1.00		
Female	3.21	1.6 - 6.31	0.001
**Place of Birth**			
Inborn	1.00		
BBA	0.23	0.08 - 0.69	0.008
Out born	0.35	0.10 - 1.20	0.096
**Resuscitation**			
No	1.00		
Yes	0.47	0.24 - 0.92	0.029
**Nasal CPAP**			
No	1.00		
Yes	4.58	1.58 - 13.31	0.005
**Hypotension**			
No	1.00		
Yes	0.05	0.01 - 0.21	< 0.001
**NEC Grade 2/3**			
No	1.00		
Yes	0.06	0.02 - 0.20	< 0.001

## Discussion

This retrospective review provides current survival rates and indicates where resources should be channeled in order to improve survival of VLBW infants in South Africa. The overall survival rate was 70.5% for VLBW infants at CMJAH 2006/2007. This is almost exactly the same as that reported from CH Baragwanath for 2000 - 2002 (71%), [22] which reflects the similarity in practice and disease profile between the two units, which form part of a single academic complex. This survival rate also compares favorably with the global average of 73% (see Table 1), but is substantially below that of developed countries [7,10,11,13,14,16]. The survival of ELBW infants in the present review of 35% was less than that in other developing countries, such as Jamaica [21] and Thailand [20] but once again very similar to that of CH Baragwanath (32%) [22]. The most significant cause of death in this study was extremely low birth weight/extreme multi-organ immaturity. This is in close agreement with national data for South Africa from the same time period - 46% of all neonatal deaths were immaturity related, of which 44.9% were due to extreme immaturity and 35.6% due to HMD [26].

The main determinants of survival in the present study - birth weight, gender, being born before arrival in the hospital, resuscitation at birth, NEC, hypotension and NCPAP are not surprising and very similar to other reports on VLBW outcome [5-7,9-22]. However, the study shows that, despite great improvements in neonatal care, the VLBW survival in South Africa is below that in other developing countries [8,9,12,15,18,20] and can improve substantially. This can be achieved by simple interventions such as ensuring preterm infants are delivered in hospital, improved neonatal resuscitation and provision of NCPAP. A provincial neonatal resuscitation programme has recently been introduced to improve the resuscitation skill of birth attendants. Patient education as to when to seek help during labour and improved emergency transport will be required to prevent preterm infant BBA. Although provision of antenatal steroids did not achieve statistical significance in this study, the number of women receiving antenatal steroids is unacceptably low (36%). This is a specific obstetric intervention that needs to be addressed and will improve neonatal outcome. Delivery by CS was advantageous, but may reflect those patients who have received antenatal care, including antenatal steroids and who delivered in hospital. It is not feasible to do elective CS on all preterm deliveries in our resource constrained setting.

South Africa is a developing country with limited health resources and high patient numbers; it is not possible to provide full tertiary support to every VLBW infant. For many years this problem has been addressed by limiting ventilatory support, including the administration of surfactant and NCPAP, to those neonates above a specified birth weight cut off. Prior to the widespread use of NCPAP, this cut off was 1000 grams. The ventilation cut off was reduced to 900 grams just before the study period, with the introduction of NCPAP to the unit. The poor survival or our ELBW infants is undoubtedly influenced by this policy and NCPAP with surfactant should be provided to babies from 750 grams in order to bring our ELBW survival rate up to that in other developing countries [12,20,21].

NEC and hypotension were the other significant predictors of mortality. The overall rate of NEC was 5.5%, which accounted for 10% of the deaths. Interestingly the rate of NEC was comparable to that in the VON in 2005, which represents well resourced settings. Prevention of NEC should also be a priority, including promotion of breastfeeding. Our rate of breastfeeding at the time of the study was extremely low, mainly due to the HIV epidemic. Only 69% of mothers were tested for HIV, but 36% were positive. The protocol during the study period was to formula feed HIV exposed babies. Of concern, is the high rate of untested mothers, the relatively high rate of refusal to be tested and failure to administer HIV prophylaxis in 16.4% of exposed mothers; this may reflect the mothers who were diagnosed after the early neonatal period. Ensuring that all mothers are counseled and tested for HIV is essential to prevent mother to child transmission and, in turn, facilitate the promotion of breastfeeding and reduction of NEC. Although 36% of screened mothers were HIV positive, HIV status did not predict neonatal outcome. This is in agreement with a study from Durban [27] which found that HIV exposed babies were not different from HIV unexposed neonates with regard to birth weight, gestational age, need for ICU admission, complications of ventilation, sepsis, IVH or death. Furthermore, most HIV exposed neonates are subsequently uninfected. HIV exposure is not a major determinant of neonatal survival and is not used as a criterion for ICU admission.

CMJAH represents a high risk obstetric population with many referrals for obstetric complications, the most frequent of which is pregnancy induced hypertension. Improved management of this obstetric complication may reduce the number of VLBW infants. There are also significant social problems with 2.6% of the babies delivered as a result of illegal termination of pregnancy and 2.3% being given up for adoption. The whole issue of preventing unwanted pregnancy is also pertinent in our population.

The low rate of in hospital screening for ROP is also an area of concern and needs to be improved. A significant number of babies are referred to the ophthalmologist at the time of their first follow up visit for ROP screening, but this may be too late in terms of adequate intervention.

## Conclusion

Although the overall survival of VLBW infants in our unit compares with the global average (see Table 1), the survival of our ELBW infants can be significantly improved. NCPAP and surfactant should be provided to ELBW infants > 750 grams birth weight. Prevention of VLBW deliveries outside the hospital, improved administration of antenatal steroids, universal screening for HIV, improved neonatal resuscitation and strategies to prevent NEC, will also improve the VLBW survival rate.

## Limitations of the study

The biggest limitation of this study is the retrospective design- this is inevitably complicated by incomplete data, lost records and diagnoses provided by different caregivers. Unfortunately, the ideal prospective collection of data by designated study personnel is extremely difficult in a developing country with limited resources (both money and manpower) where the emphasis is on service delivery.

## Abbreviations

AGA: appropriate for gestational age; BBA: born before arrival at the hospital; BPD: bronchopulmonary dysplasia; CH: Chris Hani; CMJAH: Charlotte Maxeke Johannesburg Academic Hospital; CS: Caesarean section; ELBW: extremely low birth weight infants (< 1001 grams); HIV: human immunodeficiency virus; HMD: hyaline membrane disease; IPPV: intermittent positive pressure ventilation; ICU: intensive care unit; IVH: intraventricular haemorrhage; LGA: large for gestational age; NEC: necrotising enterocolitis; NIHCD: National Institute of child health and development; NCPAP: nasal continuous positive airways pressure; PDA: patent ductus arteriousus; PVL: periventricular leukomalacia; ROP: retinopathy of prematurity; SGA: small for gestational age; VLBW: very low birth weight; VON: Vermont Oxford Network.

## Competing interests

The authors declare that they have no competing interests.

## Authors' contributions

Data collection and manuscript preparation was done by DEB; statistical analysis was done by DEB and TFC while PAC was involved in manuscript preparation. The final manuscript was approved by all authors.

## Pre-publication history

The pre-publication history for this paper can be accessed here:

http://www.biomedcentral.com/1471-2431/10/30/prepub
